# DFT Study of N_2_O Adsorption onto the Surface of M-Decorated Graphene Oxide (M = Mg, Cu or Ag)

**DOI:** 10.3390/ma12162611

**Published:** 2019-08-16

**Authors:** Zhong Liu, Xi-ren Cheng, Yi-min Yang, Hong-zhang Jia, Bao-quan Bai, Li Zhao

**Affiliations:** 1 School of Energy Power and Mechanical Engineering, North China Electric Power University, Beijing 102206, China; 2 National Engineering Laboratory for Biomass Power Generation Equipment, North China Electric Power University, Beijing 102206, China

**Keywords:** N_2_O, M-decorated graphene oxide, adsorption, density functional theory

## Abstract

In order to reduce the harm of nitrous oxide (N_2_O) on the environment, it is very important to find an effective way to capture and decompose this nitrous oxide. Based on the density functional theory (DFT), the adsorption mechanism of N_2_O on the surfaces of M-decorated (M = Mg, Cu or Ag) graphene oxide (GO) was studied in this paper. The results show that the effects of N_2_O adsorbed onto the surfaces of Mg–GO by O-end and Cu–GO by N-end are favorable among all of the adsorption types studied, whose adsorption energies are −1.40 eV and −1.47 eV, respectively. Both adsorption manners belong to chemisorption. For Ag–GO, however, both the adsorption strength and electron transfer with the N_2_O molecule are relatively weak, indicating it may not be promising for N_2_O removal. Moreover, when Gibbs free energy analyses were applied for the two adsorption types on Mg–GO by O-end and Cu–GO by N-end, it was found that the lowest temperatures required to undergo a chemisorption process are 209 °C and 338 °C, respectively. After being adsorbed onto the surface of Mg–GO by O-end, the N_2_O molecule will decompose into an N_2_ molecule and an active oxygen atom. Because of containing active oxygen atom, the structure O–Mg–GO has strong oxidizability, and can be reduced to Mg–GO. Therefore, Mg–GO can be used as a catalyst for N_2_O adsorption and decomposition. Cu–GO can be used as a candidate material for its strong adsorption to N_2_O.

## 1. Introduction

As a kind of harmful gas, Nitrous oxide can cause severe environmental problems, such as greenhouse effect, ozone depletion, acid rain and photochemical pollution [1,2,3]. Generally, it comes from biomass combustion, industrial production, selective catalytic reduction of NO_x_ as well as the nitrification and denitrification of microorganisms in soil and water [4,5]. Although the concentration of N_2_O in the atmosphere is only 322 ppb at present, its global warming potential is more than 300 times as much as that of CO_2_, because it has a long atmospheric life span of 120 years [6]. Therefore, reducing the anthropogenic emissions of N_2_O is conducive to the protection of the climate environment.

In recent years, many researches have been focused upon the adsorption and decomposition of N_2_O on various catalyst surfaces. Through the experimental studies, it is known that spinel Ni_0.75_Co_2.25_O_4_ modified with cesium cations [7], Fe/SSZ-13 [8], Co–Mn–Al mixed oxides [9] and Cu–Zn/ZnAl_2_O_4_ [10] have good catalytic effects on the N_2_O decomposition. 

Carabineiro et al. [11] also found that the order of catalytic activity of the following metal oxides for N_2_O decomposition is: Fe_2_O_3_ > CeO_2_ > ZnO > TiO_2_ > Al_2_O_3_, and doping Au into these metal oxides contributes to the reduction of an active oxygen atom on their surfaces. The decomposition of N_2_O onto the surfaces of the rare earth element (Nd, Pr, Tb and Y)-doped NiO catalysts [12] and Ag-doped Co_3_O_4_ catalysts [13] was studied, the results showing that the doping of rare earth elements or Ag can improve the catalytic activity of the catalyst. Based on the density functional theory, many researchers had investigated the adsorption and decomposition mechanisms of N_2_O on the surfaces of CaO [14], anatase TiO_2_ [15], Rh_6_ cluster [16], Ag_7_Au_6_ alloy nanocluster [17] and fullerene-like boron nitride nanocage [18]. It is found that these materials can be served as promising catalysts for N_2_O decomposing to N_2_ and a dissociated oxygen atom. Sombat et al. [19] explored the catalytic effects of metal organic structure (MOF) M_3_(BTC)_2_ (M = Fe, Cr, Co, Ni, Cu and Zn) on the oxidation of CO by N_2_O, and found that the order of the catalytic reaction rate is: Cr_3_(BTC)_2_ > Fe_3_(BTC)_2_ > Co_3_(BTC)_2_ > Ni_3_(BTC)_2_ > Cu_3_(BTC)_2_ < Zn_3_(BTC)_2_. The theoretical and experimental study of the decomposition of N_2_O on the bimetallic catalyst Rh-M (M = Co, Ni, Cu) had been carried by Hao Chen et al. [20], and it showed that the catalytic activity trend of the Rh–M catalyst is determined as Rh_7_Co_1_/SBA-15 > Rh/SBA-15 > Rh_7_Ni_1_/SBA-15 > Rh_7_Cu_1_/SBA-15. Zhang et al. found that Mg, Ce and Zn mixed with Co exhibits a good removal efficiency for N_2_O [21]. Lin et al. tested the removal efficiency of N_2_O for the RhO_x_ catalyst, results indicating that Mg doping promoted the removal of N_2_O to some extent [22]. Li et al. also found that adding MgO on Co_3_O_4_–Al_2_O_3_ had much higher and stable activity for the N_2_O decomposition compared with the Co/Al catalyst without MgO modification [23].

Due to its large specific area, high electron mobility (>200,000 cm^2^∙V^−1^∙s^−1^), high thermal conductivity (>4000 W∙m∙K^−1^) and good tensile strength, graphene has been widely used in the experimental and theoretical studies of the catalyst matrix. Many scholars have studied the catalytic decomposition of N_2_O on the surfaces of modified graphene in the last decades, and they discovered that adding Fe [24], Ga [25], Al [25,26], Se [27], Si [27,28,29], Pt [30] and ZnO [31] to modify graphene’s properties can be helpful to develop its catalytic effect upon the decomposition of N_2_O. Graphene oxide (GO) is a kind of graphene derivative, containing an oxygen functional group, which can be prepared by Hummers’ Method, and regarded as a substitute for graphene nanomaterials at low cost [32]. Lv et al. [33] have explored the adsorbing and decomposing process of our N_2_O molecule on the surfaces of Al-decorated graphene oxide (Al–GO), and they reported that the physically-adsorbed N_2_O could be decomposed to the N_2_ molecule and an O atom bonded on Al–GO exothermally (2.33 eV per N_2_O molecule), the energy barrier of which is 0.5 eV. The decomposition barrier is also decreased monotonously with the increasing electric field, and there is no barrier while the intensity of the positive electric field is 0.5 V/Å. Using the DFT computational method, Mehdi et al. [34] studied the reaction of reducing N_2_O by CO on the surfaces of the Al atom- or Si atom-decorated graphene oxide (Al–GO or Si–GO), where the results showed that the activation energy of the N_2_O decomposition process on Al–GO or Si–GO is almost negligible; the product N_2_ can be easily desorbed from the surface, which indicates that both Al–GO and Si–GO can be served as promising alternatives to enhance the N_2_O adsorption and decomposition process. 

Experimentally, graphene modified by Fe, Ga, Al, Se, Si, Pt and ZnO has been confirmed to have certain catalytic effects on the decomposition of N_2_O. Mg is a kind of active metal, and Cu and Ag are transient metals, respectively, which can be representative, and have been widely considered as potential elements for catalysts. According to previous studies, it can be concluded that Mg, Cu and Ag are promising materials for N_2_O decomposition functioning with other elements; if decorated on graphene oxide, some co-effects beneficial to N_2_O removal can be expected. However, to the best of the authors’ knowledge, there is neither any theoretical nor experimental study about the adsorption and decomposition of N_2_O onto the surfaces of Mg-, Cu- or Ag-decorated graphene oxide. The synergism of Mg, Cu or Ag combining with graphene oxide in N_2_O removal still remains unclear. In this article, the graphene oxide structure containing a single epoxy function group was selected, and Mg, Cu and Ag atoms were added for decoration, which compose the structure of Mg–GO, Cu–GO and Ag–GO. Furthermore, the adsorption of N_2_O molecules on the surface of these structures will be explored by the density functional theory.

## 2. Model and Computational Methods

We performed the geometry optimization calculation by the density functional theory (DFT) of the first principles [35,36], using the DMol^3^ module in the Materials Studio. The generalized gradient approximation (GGA) method with the Perdew-Burke-Emzerhof (PBE) function was selected for the spin unrestricted DFT-D2 computation [37,38,39,40,41]. In calculations, the double-numeric quality basis set with polarization functions (DNP) was selected, and the core treatment method DFT Semi-core Pseudopots (DSSP) was conducted. The calculation tasks were carried out with the accuracy of coarse-medium-fine, and in the fine accuracy, the convergence of energy and force were 1.0 × 10^−5^ Ha and 2.0 × 10^−3^ Ha/Å, respectively. The self-consistent field (SCF) tolerance was 1.0 × 10^−6^. In the electronic setting and density of state (DOS) calculation setting, the Brillouin zone is sampled with 8 × 8 × 1 k-points and 4 × 4 × 1 k-points under the Monkhorst-Pack scheme, respectively. The supercell of graphene was composed of 5 × 5 repeating units (containing 50 carbon atoms), where the length of crystal lattice parameters a × b × c is 12.30 Å × 12.30 Å × 25.00 Å. The length of the c direction is sufficient to eliminate the effect of the pseudopotential interaction between the adjacent cell systems. The computational formula of the adsorption energy for adsorbate (A) adsorbed on adsorbent (B) is E_ads_ (A) = E (A + B) − E (A) − E (B), in the formula, E (A + B), E (A) and E (B) is the total energy of adsorption structure of A adsorbed on B, adsorbate (A) and adsorbent (B) at 0 K, respectively. The basis set superposition error (BSSE) was not considered, since previous studies have shown that the numerical basis sets implemented in DMol^3^ can minimize or even eliminate BSSE [28,41,42]. 

## 3. Results and Discussions

### 3.1. N_2_O Adsorbed on the Surface of GO

**Figure 1:**
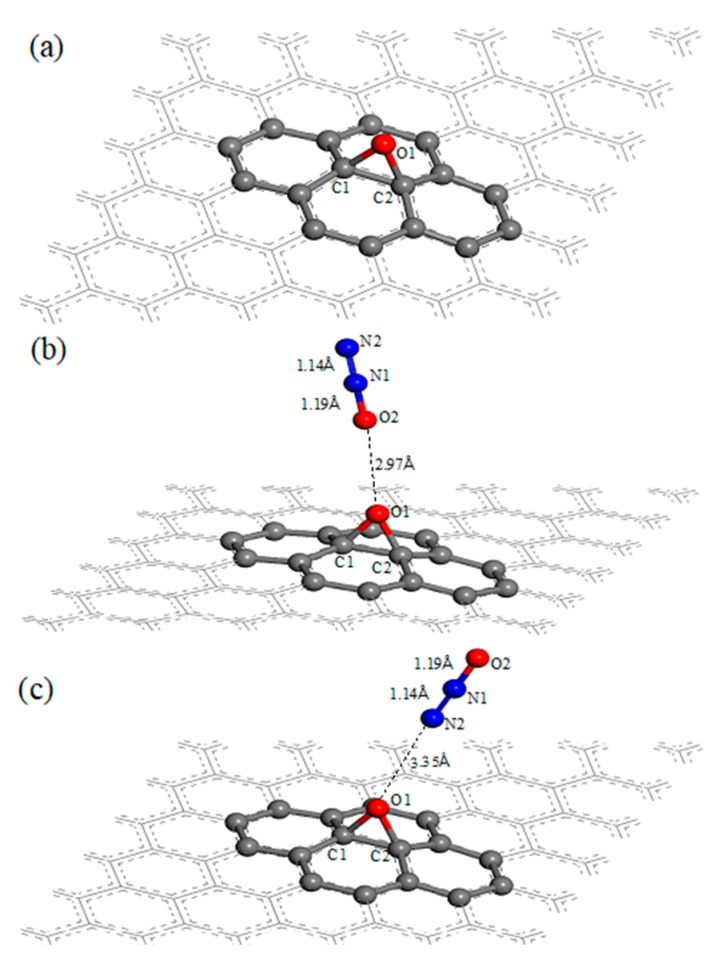
Structure of (**a**) graphene oxide (GO) and nitrous oxide (N_2_O) adsorbed on the surface of GO by (**b**) O-end and (**c**) N-end.

The graphene oxide structure in this article contains an epoxy functional group, as showed in Figure 1a. The oxygen atom (O1) was located above the center of the bond of two neighboring carbon atoms (C1, C2), the lengths of O1–C1 bond and O1–C2 bond are both 1.46 Å, the bond angle of ∠O1–C1–C2 and ∠O1–C2–C1 are both 58.7° (listed in Table 1). Compared with the primary graphene, the carbon atoms C1 and C2, which are under the oxygen atom O1 in the graphene oxide, are protruded from the plane of the primary graphene, while the distance between the two atoms and the plane is 0.42 Å. The length of C1–C2 bond is 1.51 Å, which is larger than the carbon–carbon bond length (1.42 Å) in the primary graphene. Figure 1b,c are the structural diagrams of an N_2_O molecule adsorbed on the surface of graphene oxide through O-end and N-end, respectively. In Figure 1b, the distance between the N_2_O molecule and oxygen atom (O1) is 2.97 Å, and the adsorption energy of this N_2_O molecule adsorbed on the surface of graphene oxide is −0.06 eV, while the distance is 3.35 Å and the adsorption energy is −0.03 eV in Figure 1c. It shows that the adsorption of the N_2_O molecule on the surface of graphene oxide by O-end is slightly stronger than by N-end, and both belong to physisorption. These adsorption energy values are lower than that of N_2_O molecules adsorbed on the surface of primary graphene, which is −0.07 eV [25]; this phenomenon indicates that the oxygen atom (O1) on graphene oxide weakens the interaction between the N_2_O molecule and graphene surface. The Hirshfeld charges of N_2_O, GO, N_2_O–GO (O-end) and N_2_O–GO (N-end) are listed in Table 2; the latter two are the structures of N_2_O molecules adsorbed onto the surface of graphene oxide through O-end and N-end. It can be found that the charge of each atom in N_2_O and GO does not change obviously before and after the adsorption of N_2_O on the surface of graphene oxide by O-end or N-end, which indicates that there is no charge transfer between N_2_O and GO, and means that the interaction between them is weak.

### 3.2. Mg-, Cu- and Ag-Decorated Graphene Oxide

**Figure 2:**
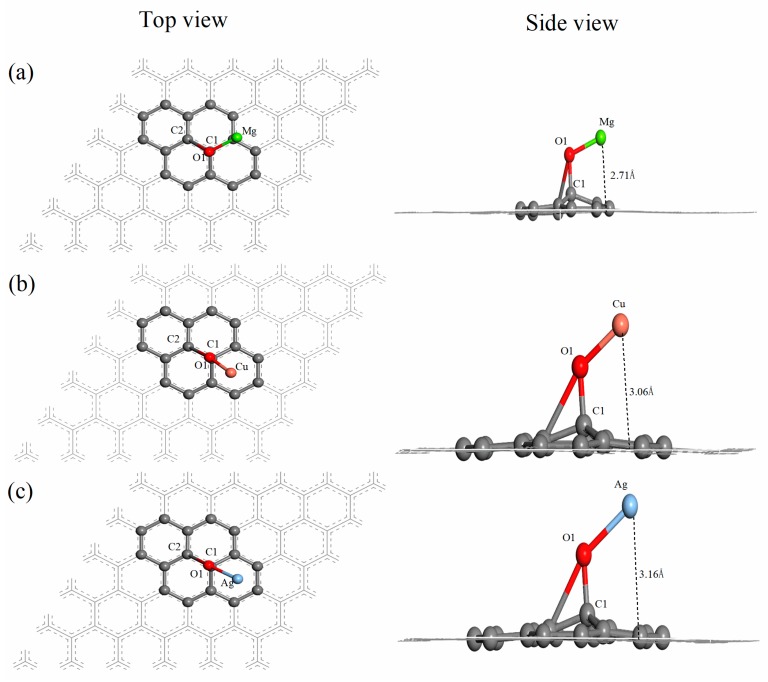
Top view and side view of the optimized structure of Mg–GO (**a**), Cu–GO (**b**) and Ag–GO (**c**).

The top view and side view of the structures of the Mg, Cu and Ag atom-modified graphene oxide (Mg–GO, Cu–GO and Ag–GO) are shown in Figure 2. The detailed structural parameters of Mg–GO, Cu–GO and Ag–GO are listed in Table 1. The bond length of the C1–O1 bond in Mg–GO, Cu–GO and Ag–GO is 1.47 Å, 1.47 Å and 1.45 Å, respectively, which indicates that the differences between bond lengths are small. However, the bond angle of ∠O1–C1–C2 increases from 58.7° to 100°, and the distance between the oxygen atom O1 and the carbon atom C2 is greater than 2.26 Å, indicating that after Mg, Cu or Ag doping, the C2–O1 bond breaks on the surface of graphene oxide. Moreover, the oxygen atom O1 shifts from the top of the center of our C1–C2 bond to the top of the carbon atom C1. Thus, the C1–O1 bond becomes almost perpendicular from inclining to the graphene plane. The lengths of the Mg–O1 bond in Mg–GO, the Cu–O1 bond in Cu–GO, and the Ag–O1 bond in Ag–GO are 1.87 Å, 1.80 Å, and 2.10 Å, respectively. The first two are similar to the length of the Fe–O bond in Fe–GO (1.83 Å), but longer than the length of the Al–O bond in Al–GO (1.70 Å) and the Si–O bond in Si–GO (1.70 Å) [42]. It is clear that the interaction between Ag and O in Ag–GO is the weakest among these metal atoms-decorated graphene oxide, since the length of Ag–O1 bond is the longest. The bond angles of ∠C1–O1–Mg, ∠C1–O1–Cu and ∠C1–O1-Ag are 106.7°, 120.4° and 159.4°, respectively. From the top view in Figure 2, it can be seen that the angle between the Mg–O1 bond and C1–C2 bond is approximately 120°, while the angle between the Cu–O1 bond (or Ag–O1 bond) and C1–C2 bond is approximately 180°. In the side view of Figure 2, the vertical distances between the Mg, Cu and Ag atoms and the graphene plane are marked as 2.71 Å, 3.06 Å and 3.16 Å, respectively.

The adsorption energies of Mg, Cu and Ag atoms adsorbed on the surface of GO are −1.76 eV, −1.84 eV and −1.13 eV, respectively, all belonging to chemisorption. The Hirshfeld charges of atoms or the partial structure in Mg–GO, Cu–GO and Ag–GO are listed in Table S1 in Supplementary Information. It can be found that the charges of the oxygen atom and graphene part in GO increase after doping the Mg, Cu and Ag atoms. Although the adsorption energy of Mg adsorbed on GO and the distance between the Mg atom and O1 atom are both slightly lower than those of Cu, the interaction between the Mg atom and the graphene surface is the strongest among the three metal atoms, because the distance between Mg and the graphene surface is the shortest, and the charge transfer after Mg doping is the strongest.

**Figure 3:**
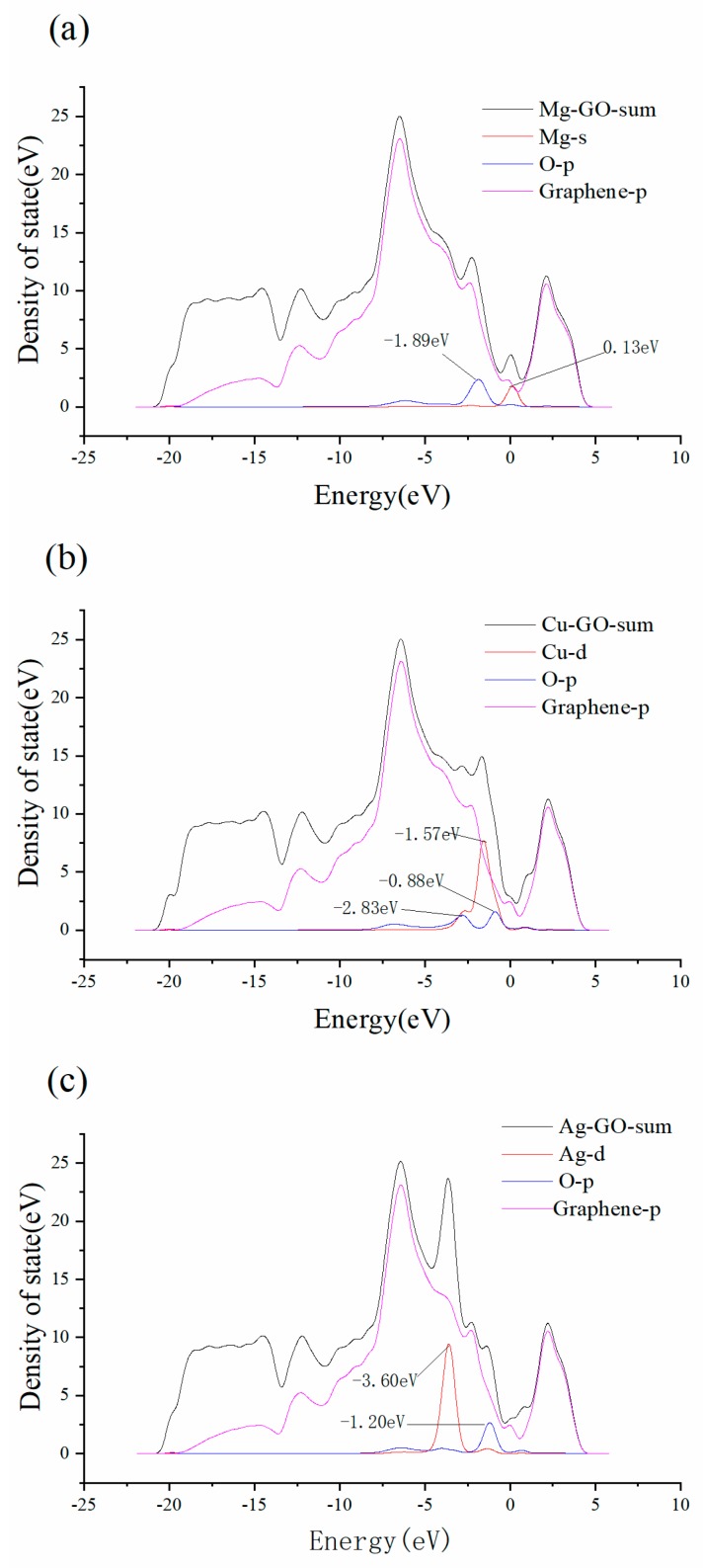
Local density of state (LDOS) of Mg–GO (**a**), Cu–GO (**b**) and Ag–GO (**c**).

Figure 3a is the local density of state (LDOS) graph of the Mg–GO structure. It can be seen that there are two peaks at 0.13 eV higher than the Fermi energy for Mg-s (red) and −1.89 eV lower than Fermi energy for O-p (blue). 

Both the two peaks contribute to the peak of the corresponding region of the total state density Mg–GO-sum (black), indicating the 3s bands for Mg and 2p bands for the oxygen atom O1 are well bound to yield a set of hybridized p bands, which leads to the formation of an Mg–O1 bond with a length of 1.87 Å. Figure 3b is the LDOS of the Cu–GO structure. There are two peaks at approximately −2.83 eV and −0.88 eV below the Fermi energy for O-p (blue) and a peak at −1.57 eV below the Fermi energy for Cu-d (red). All the three peaks contribute to the peak of the corresponding region of the total density of state density Cu–GO-sum (black), which shows that 3d bands for Cu and 2p bands for the oxygen atom O1 are well hybridized, which results in the formation of the Cu–O1 bond with a length of 1.80Å. Figure 3c is the LDOS of the Ag–GO structure. It can be observed that there is a peak at −3.60 eV below the Fermi energy for Ag-d (red), where a peak appears at the corresponding region for the total density of state density Ag–GO-sum (black). However, there is a peak at −1.20 eV below the Fermi energy for O-p (blue), where no clear peak appears for Ag–GO-sum (black). It indicates that the interaction between Ag and the oxygen atom O1 in GO is not strong. 

### 3.3. N_2_O Adsorbed on the Surface of M-GO (M = Mg, Cu or Ag)

**Figure 4:**
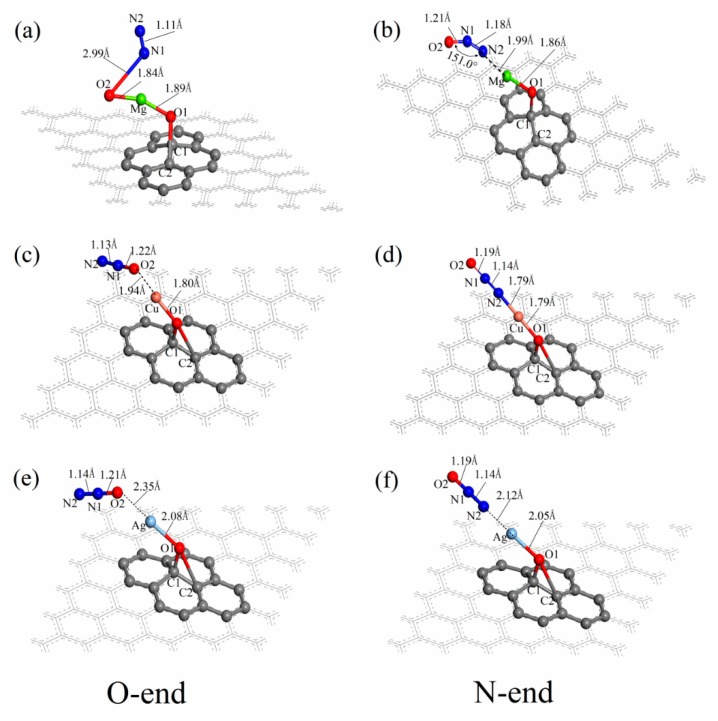
The adsorption structures of N_2_O adsorbed on the surface of Mg–GO (**a**,**b**), Cu–GO (**c**,**d**) and Ag–GO (**e**,**f**) by O-end or N-end.

The adsorption structures of an N_2_O molecule on the surfaces of Mg–GO, Cu–GO and Ag–GO by O-end or N-end had been optimized, and the final structure is shown in Figure 4. It can be seen from Figure 4a that the structure of the N_2_O molecule has obvious changes after being adsorbed on the surface of Mg–GO by O-end. The distance between the oxygen atom O2 and the nitrogen atom N1 in the N_2_O molecule increases from 1.19 Å to 2.99 Å, the N1–O2 bond breaks; the distance between the nitrogen atoms N1 and N2 shortens from 1.14 Å to 1.11 Å, which is close to the length of the triple bond in the nitrogen molecule. It indicates that our N_2_O molecule is decomposed into an active oxygen atom O2 and an N2 molecule, while being adsorbed on the surface of Mg–GO by O-end. The distance between the oxygen atom O2 and Mg atoms is 1.84 Å, and the length of the Mg–O1 bond in Mg–GO elongates a little, from 1.87 Å to 1.89 Å. Figure 4b is the structure of the N_2_O molecule adsorbed on the surface of Mg–GO by N-end. As the graph has shown, the lengths of this N1–O2 bond and N1–N2 bond in the N_2_O molecule both elongate slightly, are 1.21 Å and 1.18 Å, respectively. The angle of ∠N2–N1–O2 changes from 180.0° to 151.0°, and the molecular configuration type is transformed from linear to V-shaped. The distance between the N_2_O molecule and Mg atom in Mg–GO is 1.99 Å. 

The graphs c, d, e and f in Figure 4 are the structures of N_2_O molecules adsorbed on the surface of Cu–GO and Ag–GO by O-end and N-end respectively. It can be seen from these graphs that there is no obvious change for the N1–O2 bond and N1–N2 bond in the N_2_O molecule, whether it is absorbed by O-end or N-end on the surfaces of Cu–GO and Ag–GO, and the molecular configuration is still linear. The distances between the N_2_O molecule and Cu atom in Cu–GO are respectively 1.94 Å and 1.79 Å, while N_2_O is adsorbed on the surface of Cu–GO by O-end and N-end. The distances between the N_2_O molecule and the Ag atom in Ag–GO are 2.35 Å and 2.12 Å, respectively, while N_2_O is adsorbed on the surface of Ag–GO by O-end and N-end. It can be observed in the Figure 4d that the N_2_O molecule and Cu atom in Cu–GO are roughly in a line after the N_2_O molecule adsorbed on the surfaces of Cu–GO by N-end.

The Hirshfeld charges of the six adsorption structures N_2_O–Mg–GO(O-end), N_2_O–Mg–GO(N-end), N_2_O–Cu–GO (O-end), N_2_O–Cu–GO (N-end), N_2_O–Ag–GO (O-end) and N_2_O–Ag–GO (N-end) are listed in the Table S1. Comparing the Hirshfeld charges of N_2_O, Mg–GO and N_2_O–Mg–GO (O-end), it can be found that after N_2_O adsorbed on the surface of Mg–GO by O-end, the charge of the oxygen atom O2 becomes −0.492 e from −0.109 e, and the charges of nitrogen atoms N1 and N2 changes from 0.192 e and −0.083 e to 0.065 e and 0.055 e. The charge of the N_2_O part changes from 0 e to −0.371 e, and charge of the graphene part changes from −0.173 e to 0.204 e, the losing charge amount of which is 0.377 e, while the charge changes of the Mg atom and oxygen atom O1 in Mg–GO are −0.017 e and 0.012 e, respectively, which indicates that the charge is mainly transferred from the graphene surface to the oxygen atom O2 of the N_2_O molecule, and breaking the N1–O2 bond. According to the Hirshfeld charges of N_2_O, Mg–GO and N_2_O–Mg–GO(N-end), it can be seen that the variations of the charges of the O2, N1 and N2 in N_2_O molecule are 0.021 e, −0.036 e and −0.066 e, respectively. The charge changes of N_2_O and the Mg atom are −0.081 e and 0.105 e, respectively, while charge changes of the graphene part and oxygen atom O1 are both −0.012 e. Thus, only part of the charges from this Mg atom are transferred to the N_2_O molecule when the N_2_O molecule is adsorbed on the surface of Mg–GO by N-end. 

By comparing and analyzing the Hirshfeld charges of N_2_O, Cu–GO, N_2_O–Cu–GO(O-end) and N_2_O–Cu–GO (N-end), it can be concluded that the charges of the N_2_O molecule are both transferred to the Cu atom and graphene part, while the N_2_O molecule is adsorbed on the surface of Cu–GO through O-end and N-end. 

For O-end, the charge transfer amounts of the former are −0.103 e and −0.048 e, and for N-end, those are −0.048 e and −0.018 e; the former are apparently larger than the latter. By analyzing the Hirshfeld charges of N_2_O, Ag–GO, N_2_O–Ag–GO (O-end) and N_2_O–Ag–GO (N-end), it can be seen that while being adsorbed on the surface of Ag–GO by O-end and N-end, the N_2_O molecule loses charges of 0.13 e or so, 0.1 e of which are transferred to the graphene part, and the rest to the oxygen atom O1, and there is no obvious change for the charges of the Ag atom.

The adsorption energies of the N_2_O molecule adsorbed on the surfaces of graphene, GO, Mg–GO, Cu–GO and Ag–GO through O-end or N-end are listed in Table 2. As is shown, all the adsorption energy absolute values of N_2_O molecule adsorbed on the surfaces of Mg–GO, Cu–GO and Ag–GO are larger than 0.4 eV, and belong to chemisorption, which are much stronger than the N_2_O adsorption on the surfaces of primary graphene and graphene oxide. It indicates that the addition of metal atoms Mg, Cu or Ag on the surface of graphene oxide can improve the adsorption of the N_2_O molecule on the surface of graphene oxide. The adsorption energy of this N_2_O molecule adsorbed on the surface of Mg–GO by O-end is close to that of an N_2_O molecule adsorbed on the surface of Cu–GO by N-end, as can be inferred from Table 2. Compared with on the surfaces of Mg–GO and Cu–GO, the adsorptions of N_2_O on the surface of Ag–GO are clearly much weaker. The adsorption of N_2_O on the surface of Ag–GO through O-end belongs to weak chemical adsorption, and the adsorption energy value of N_2_O on the surface of Ag–GO via N-end is also lower than that of on Mg–GO or Cu–GO, which shows that the effect of adding an Ag atom on the surface of graphene oxide is not as good as adding an Mg atom and Cu atom. Furthermore, according to previous DFT calculations, the adsorption energies of N_2_O on GO decorated by Fe, Ga, Al, Se, Si, Pt and ZnO are −1.07 eV, −0.27 eV, −0.65 eV, −0.22 eV, −0.18 eV, −0.33 eV and −0.27 eV, respectively. Therefore, it can be concluded that the adsorption of N_2_O on Mg–GO and Cu–GO can be relatively stable due to its large adsorption energy. For Ag–GO, the adsorption is relatively weak.

**Figure 5:**
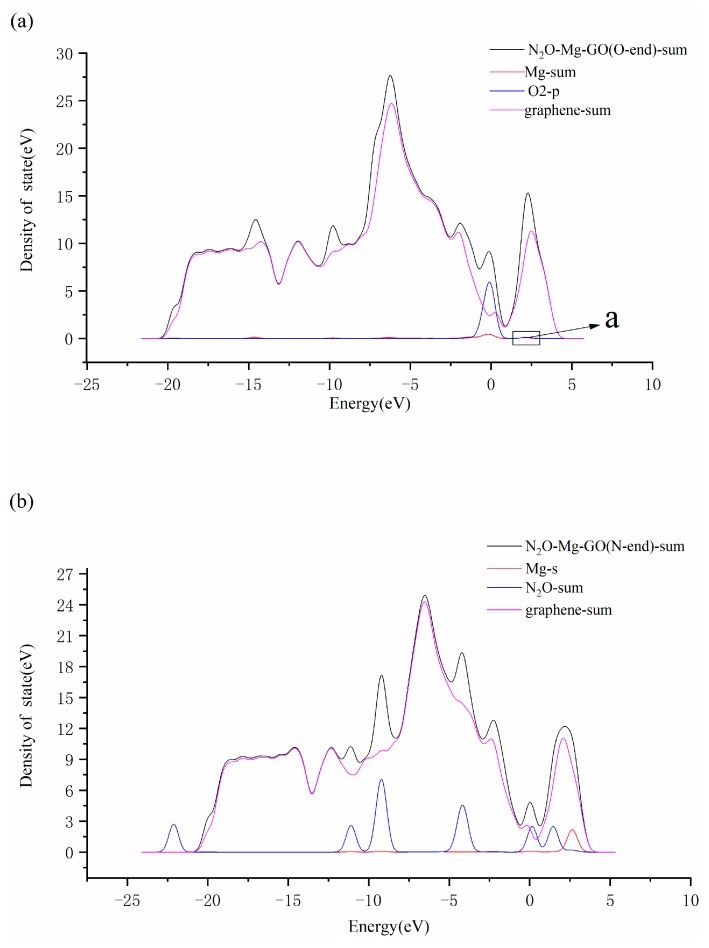
LDOS of N_2_O–Mg–GO (O-end) (**a**) and N_2_O–Mg–GO (N-end) (**b**).(The enlarged part a of Figure 5 is shown in the Supplementary Information 1.)

Figure 5a,b are the LDOS of the structures N_2_O–Mg–GO (O-end) and N_2_O–Mg–GO (N-end), respectively. As shown in Figure 5a, there are two overlapped peaks near the Fermi level and in the region of 1.8–2.8 eV higher than Fermi level, for Mg-sum and O2-p. It indicates that the 3s bands for the Mg atom and 2p bands for the oxygen atom O2 in N_2_O are well hybridized, which leads to the formation of the Mg–O2 bond with a length of 1.84 Å. However, there is no obvious overlapping peak between the DOS curves for Mg-s and N_2_O-p in Figure 5b, indicating that no strong interaction appears between the Mg atom and the terminal nitrogen atom N2 in the N_2_O molecule. 

**Figure 6:**
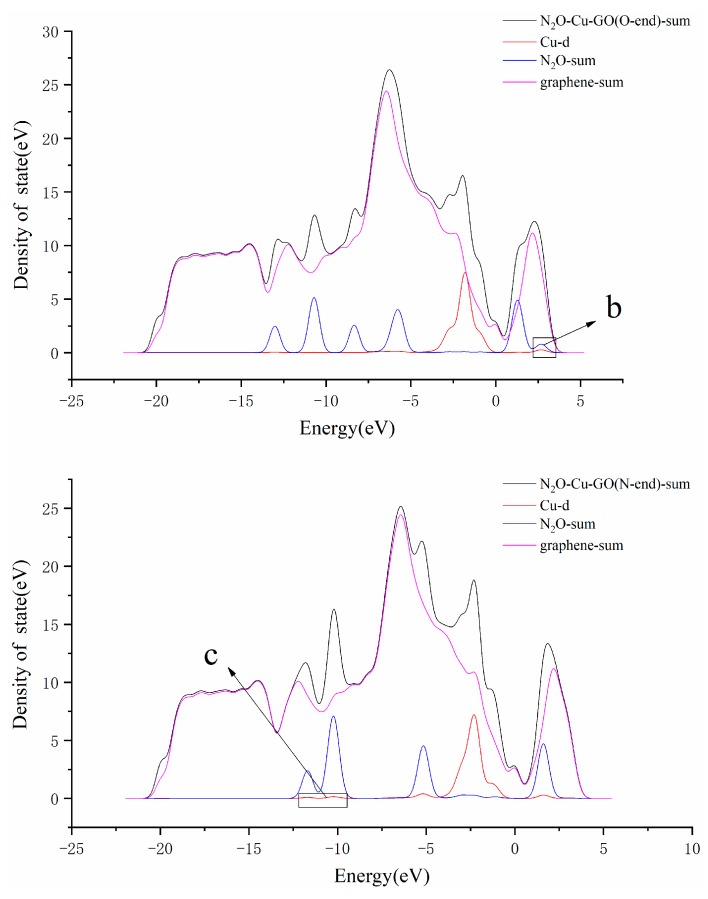
LDOS of N_2_O–Cu–GO (O-end) (**a**) and N_2_O–Cu–GO (N-end) (**b**).(The enlarged part b and c of Figure 6 are shown in the Supplementary Information 2 and 3, especially.)

The local density of states of N_2_O–Cu–GO (O-end) and N_2_O–Cu–GO (N-end) are showed in Figure 6. It can be seen that there is only an overlapping peak for the DOS curves of Cu-d and N_2_O-sum, which is at the region of 1.8–2.7 eV higher than Fermi level, in Figure 6a. While in Figure 6b, it can be observed that there are four overlapping peaks for the DOS curves of Cu-d and N_2_O-sum, two of which are in the region of −12.0~−10.0 eV lower than the Fermi level, while the others are near −5.0 eV lower than and 1.6 eV higher than the Fermi level, respectively. It can be seen that the 3d bands for Cu and the p bands for N_2_O hybrid well, contributing to the formation of a Cu–N2 bond with a length of 1.79 Å. It indicates that the adsorption of N_2_O on the surface of Cu–GO by N-end is stronger than that by O-end, and the reason that the adsorption value of the former is larger than that of latter is also explained. 

**Figure 7:**
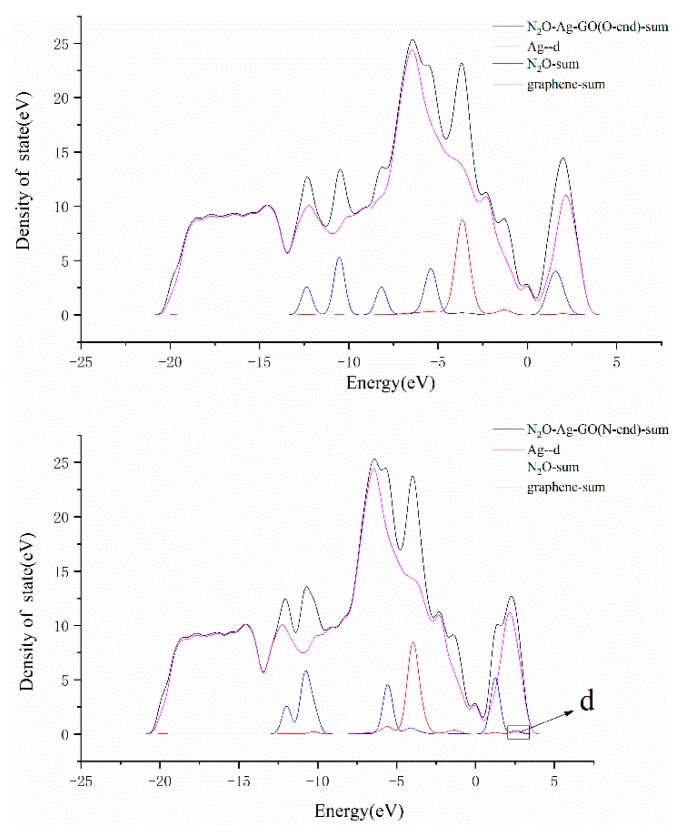
LDOS of N_2_O–Ag–GO (O-end) (**a**) and N_2_O–Ag–GO (N-end) (**b**).(The enlarged part d of Figure 7 is shown in the Supplementary Information 4.)

The graphs in Figure 7 are the LDOS of the structures N_2_O–Ag–GO (O-end) and N_2_O–Ag–GO (N-end). Observing the DOS curves of Ag-d and N_2_O-sum, it can be found that there are two overlapping peaks near −5.5 eV lower than the Fermi level and in the region of 2.2~2.8 eV, respectively, which are higher than the Fermi level in Figure 7a. Whereas, no overlapping peak is found in Figure 7b. It shows that that the adsorption of N_2_O on the surface of Ag–GO by N-end is stronger than that by O-end, and also explains the reason why the distance between N_2_O and Ag atoms is closer and the adsorption energy is larger when the N_2_O molecule is adsorbed on the surface of Ag–GO by N-end. 

According to above discussion, it can be concluded that the N_2_O molecule decomposes into N_2_ and an active oxygen atom O, after being adsorbed on the surface of Mg–GO by O-end. It is the interaction between the N_2_O molecule and the Mg atom of the GO system that activates the N_2_O molecule and catalyzes the decomposition of N_2_O. The desorption energy of N_2_ from the structure N_2_–O–Mg–GO is −0.32 eV, and the deformed structure O–Mg–GO has strong oxidizability for the active oxygen atom O, which can be reduced to Mg–GO by reductants, such as CO. The adsorption energy is the highest and the distance between N_2_O and GO is the nearest. Thus, Mg–GO can be used as a kind of cyclic catalyst for N_2_O adsorption and decomposition.

### 3.4. Gibbs Free Energy Analysis

**Figure 8:**
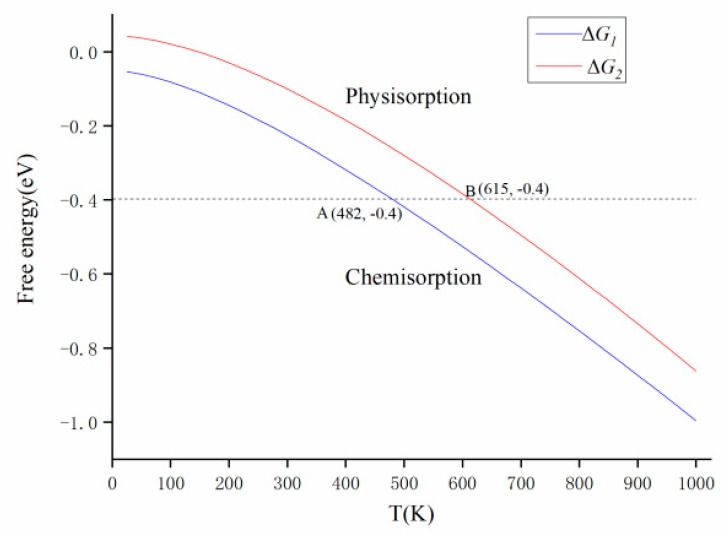
Gibbs free energy of N_2_O adsorbed on the surface of Mg–GO by O-end (*∆G*_1_, blue line) and Cu–GO by N-end (*∆G*_2_, red line).

The above analysis showed that the probability of having an N_2_O molecule adsorbed on the surface of Mg–GO via O-end is larger than that via N-end, while on the surface of Cu–GO, an N_2_O molecule being adsorbed by the N-end is more likely to occur. The Gibbs free energies of N_2_O adsorbed on the surfaces of Mg–GO through O-end and Cu–GO through N-end were calculated. The computational formula is: *∆G*_1_ (T) = *G*_N2O-Mg-GO_ (T) − *G*_Mg-GO_ (T) − *G*_N2O_ (T) and *∆G*_2_ (T) = *G*_N2O-Cu-GO_ (T) − *G*_Cu-GO_ (T) − *G*_N2O_ (T). *∆G*_1_ (T) and *∆G*_2_ (T) are the Gibbs free energies of the adsorption of N_2_O on the surfaces of Mg–GO through the O-end and Cu–GO through the N-end at the temperature of T, respectively. *G*_N2O-Mg-GO_ (T), *G*_N2O-Cu-GO_ (T), *G*_Mg-GO_ (T), *G*_Cu-GO_ (T) and *G*_N2O_ (T) are Gibbs the free energies of the structures N_2_O–Mg–GO (O-end), N_2_O–Cu–GO (N-end), Mg–GO, Cu–GO and N_2_O at the temperature of T, respectively. The Gibbs free energy formula is *G* (T) = *H* (T) – T × *S* (T), *H* (T) and *S* (T) are enthalpy and entropy at the temperature of T, respectively. The calculation results are shown in Figure 8. When│*∆G*│> 0.4 eV, the adsorption of N_2_O on the surface is strong, which could be classified as chemisorption; when│*∆G*│< 0.4 eV, the adsorption of N_2_O on the surface is weak, which could be seen as physisorption. From Figure 8, it can be found that when the temperature T1 > 482 K = 209 °C, and the Gibbs free energy│*∆G*_1_│> 0.4 eV, the adsorption of N_2_O on the surface of Mg–GO by the O-end is a chemisorption reaction. While for the adsorption of N_2_O on the surface of Cu–GO by N-end, the temperature T2 > 615 K = 338 °C, and the Gibbs free energy│*∆G*_2_│> 0.4 eV. It shows that the temperature range, in which the chemisorption of N_2_O on the surface of Mg–GO can successfully occur, is larger than that of Cu–GO. 

## 4. Conclusions

In this article, the adsorption of an N_2_O molecule on the surfaces of Mg-, Cu- or Ag-modified graphene oxide by O-end or N-end had been studied with the density functional theory. The results show that Mg, Cu and Ag modification have different effects for N_2_O catalytic decomposition. Compared to N-end, the adsorption of the N_2_O molecule on the surface of Mg–GO by O-end is more likely to occur, whose adsorption energy is −1.47 eV. After adsorption, an N_2_O molecule will decompose into an N_2_ molecule and an active oxygen atom, the latter is in the structure O–Mg–GO, which can be reduced by reductants to Mg–GO. While on the surface of Cu–GO, the N_2_O molecule is more likely to be adsorbed through the N-end, the adsorption energy is −1.40 eV, and there is no change for the configuration of this N_2_O molecule during the adsorption. According the Gibbs free energy analysis, the minimum temperature for the chemisorption of N_2_O on the surface of Mg–GO by O-end is 209 °C, and that of Cu–GO by the N-end is 338 °C. Therefore, Mg–GO can be used as a catalyst for N_2_O adsorption and decomposition. Cu–GO can be used as a candidate material for its strong adsorption to N_2_O. 

## Figures and Tables

**Table 1:** Structural parameters of GO and M–GO (M = Mg, Cu or Ag).

System	Bond Length/Å			Bond Angle/°		
	C1–O1	C2–O1	M–O1	∠C2–C1–O1	∠C1–C2–O1	∠C1–O1–M
**GO**	1.46	1.46	-	58.7	58.7	-
**Mg–GO**	1.47	2.35	1.87	105.0	37.0	106.7
**Cu–GO**	1.47	2.28	1.80	100.3	39.4	120.4
**Ag–GO**	1.45	2.26	2.10	100.1	39.1	159.4

**Table 2:** Adsorption energy of N_2_O on the surface of graphene, GO and M–GO (M = Ag, Mg or Cu).

System		Graphene	GO	Mg–GO	Cu–GO	Ag–GO
**N_2_O adsorption energy/eV**	**O-end**	−0.07 [33]	−0.06	−1.40	−0.82	−0.45
	**N-end**	−0.07 [33]	−0.03	−0.83	−1.47	−0.66

## Supplementary Materials

The following are available online at https://www.mdpi.com/1996-1944/12/16/2611/s1, Figure S1: LDOS of N_2_O–Mg–GO (O-end), Figure S2: LDOS of N_2_O–Cu–GO (O-end), Figure S3: LDOS of N_2_O–Cu–GO (N-end), Figure S4: LDOS of N_2_O–Ag–GO (N-end), Table S1: Hirshfeld charges of atoms or partial structure in those systems below (M = Mg, Cu or Ag).
