# Supplementary material for: DFT Study of N2O Adsorption onto the Surface of M-Decorated Graphene Oxide (M = Mg, Cu or Ag)
Source: Materials (Basel). 2019 Aug 16;12(16):2611. doi: 10.3390/ma12162611 (PMC6720633; doi:10.3390/ma12162611)
Supplement: Supplementary file 1 [file materials-12-02611-s001.pdf]

materials

MDPI

Article

DFT Study of N_2O Adsorption onto the Surface of M-Decorated Graphene Oxide (M = Mg, Cu or Ag)

Zhong Liu [1], Xi-ren Cheng [1], Yi-min Yang [1], Hong-zhang Jia [1], Bao-quan Bai [1] and Li Zhao [2,*]

Supplementary information 1:

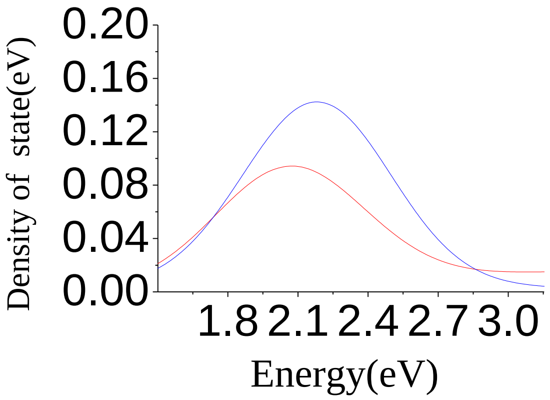

Figure S1. LDOS of N_2O–Mg–GO (O-end).

Supplementary information 2:

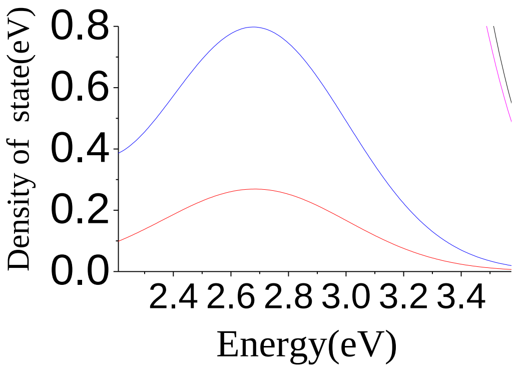

Figure S2. LDOS of N_2O–Cu–GO (O-end).

Supplementary information 3:

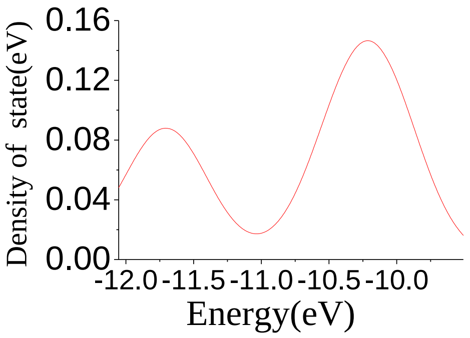

Figure S3. LDOS of N_2O–Cu–GO (N-end).

Supplementary information 4:

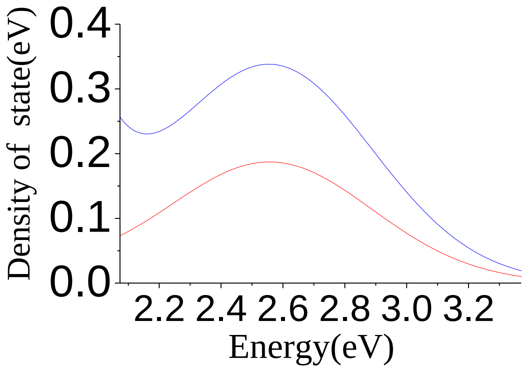

Figure S4. LDOS of N_2O–Ag–GO (N-end).

Supplementary information 5:

Table S1. Hirshfeld charges of atoms or partial structure in those systems below (M = Mg, Cu or Ag)[a].

System	C1	C2	O1	M	O2	N1	N2	N2O	Graphene
**N_2O**	-	-	-	-	−0.109	0.192	−0.083	0	-
**GO**	0.052	0.052	−0.133	-	-	-	-	-	0.133
**N_2O–GO (O-end)**	0.051	0.051	−0.133	-	−0.103	0.192	−0.087	0.02	0.131
**N_2O–GO (N-end)**	0.052	0.052	−0.126	-	−0.115	0.189	−0.075	−0.01	0.127
**Mg–GO**	0.046	−0.007	−0.343	0.516	-	-	-	-	−0.173
**Cu–GO**	0.039	0.002	−0.276	0.336	-	-	-	-	−0.06
**Ag–GO**	0.045	−0.001	−0.262	0.293	-	-	-	-	−0.031
**N_2O–Mg–GO (O-end)**	0.051	0.001	−0.331	0.499	−0.492	0.065	0.055	−0.372	0.204
**N_2O–Mg–GO (N-end)**	0.044	−0.008	−0.355	0.621	−0.088	0.156	−0.149	−0.081	−0.185
**N_2O–Cu–GO (O-end)**	0.04	−0.001	−0.289	0.233	−0.065	0.216	0.013	0.164	−0.108
**N_2O–Cu–GO (N-end)**	0.044	−0.002	−0.295	0.288	−0.066	0.214	−0.063	0.085	−0.078
**N_2O–Ag–GO (O-end)**	0.039	0.001	−0.29	0.293	−0.082	0.213	−0.002	0.129	−0.132
**N_2O–Ag–GO (N-end)**	0.039	0	−0.298	0.301	−0.053	0.227	−0.047	0.127	−0.13

[a] negative sign implies electrons gained, whereas a positive sign implies electrons lost by the atom (unit of charge is electron).

© 2019 by the authors. Submitted for possible open access publication under the terms and conditions of the Creative Commons Attribution (CC BY) license (http://creativecommons.org/licenses/by/4.0/).
